# 

**Published:** 1911-05-13

**Authors:** 


### Jottings.

On January 25 his Excellency the Governor of New South Wales formally opened the new additions to the Berrima District Hospital, which have been carried out at a cost of £1,250.

The corner-stone of the new hospital in Toronto, Canada, which, when completed, will be the finest structure of the kind in the Dominion, was laid by Lord Grey, Governor-General, on April 11 in the presence of a number of distinguished guests. The hospital will cost half a million sterling.

The hospital commission of the town of Berlin has decided to suppress the custom of indicating on the head-card of patients in the hospitals the name of the disease from which they are suffering. In surgical wards the disease will in future be indicated by the first syllable of its Latin name.

### Gifts and Bequests.

By the will of Mr. Henry Hill the Worcester Ophthalmic Hospital receives £3,000.

Mrs. Jessie Mitchell, of Kilmarnock, has left £1,000 to the Kilmarnock Infirmary.

Miss Fanny Harman, of Woking, has left £2,000 to the Royal Berkshire Hospital, Reading.

Mr. Frederick Bond, of Bournemouth, has left £1,000 to the National Anti-Vivisection Society.

Mr. George Stratford Hunt, of Chislehurst, has bequeathed £100 to the Bromley Cottage Hospital.

Mr. John Try-Davies, of Hyde Park, has left the residue of his estate upon trust for Robert Lindsay for life, with remainder to the Montreal General Hospital.

Prince Alexander of Teck has received £72 13s. which has been collected by Princess Mary on behalf of the Prince Francis of Teck Memorial Fund for the Middlesex Hospital.

Mr. Holmes Wright, of Bingley, Yorks, press paper manufacturer, who has left estate of the gross value of £303,977, bequeathed £1,000 to the Bingley Cottage Hospital, and £500 to the Royal Asylum for Idiots, Lancaster.

Mr. Frederic Abraham Phillips, of Inverness Terrace, W., has bequeathed £1,000 to his wife absolutely, requesting (but without creating any trust in that respect) that she will distribute the same in sums not exceeding £50 to such hospitals in England and such Jewish charities as she may select.

Mrs. Elizabeth Timm, of Bryanston Square, W., has bequeathed £1,000 each to University College Hospital, Gower Street, St. Mary's Hospital, Paddington, Paddington Green Children's Hospital, and to the Middlesex Hospital; £200 to the Samaritan Hospital for Women, Marylebone Road; and £100 to the Western General Dispensary, Stafford Street, Marylebone.

Two thousand guineas have been handed over to the Beckett Hospital Governors, being another record contribution from the Barnsley and District Hospital Saturday and Sunday Committee. The Barnsley Cordwainers' Society has granted £200 to the Building Fund, and legacies of £100 and £25 respectively have been received from Mrs. Race and Mrs. Maddison.

Mr. Charles Wertheimer, who died on Tuesday last week, has left subject to certain conditions the ultimate residue of his estate for division between the following, among other charities: Four-twentieths to the London Hospital; two-twentieths to the Evelina Hospital; one-twentieth to the Royal London Ophthalmic Hospital; and one-twentieth to St. Thomas's Hospital. The unrivalled art collections of the testator will, under the terms of the will, come into the market.

Mrs. Emily Tatchell, of Holland Park, W., and of the Isle of Wight, has left £100 each to the Hospital for Consumption and Diseases of the Chest, Fulham Road, S.W., the Free Cancer Hospital, Fulham Road, S.W., the National Hospital for Diseases of the Heart, Soho Square, W.C., the Home for the Blind, Alma Square, St. John's Wood, N.W., the Samaritan Free Hospital for Women and Children, Marylebone Road, N.W., Queen Charlotte's Lying-in Hospital, Marylebone Road, St. Mary's Hospital, Paddington, the Middlesex Hospital, the Chelsea Hospital for Women, the Hospital for Diseases of the Throat, Nose, and Ear, Golden Square, and the Dental Hospital, Leicester Square.

### THE COMPLETE WARD UNIT.

"A Hospital Superintendent" writes as follows: In your issue of May 6 a plan is reproduced under the heading of "A Complete Ward Unit for a Modern Hospital," and in your *résumé* of the Week's Work you invite any criticism on the plan which is reproduced. Before offering any criticism it would be necessary to know the relationship of the other ward units in the hospital to the one illustrated. A complete ward unit would naturally include at least one male and one female ward with their annexes. I am sure your readers would be indebted to the gentlemen who have furnished the plan and description in this week's issue if they would respond to this request. Perhaps in their description they would indicate how the nursing is carried on in the single ward behind the kitchen. Do bed-pans require to be carried through the four-bedded ward to the lavatory, or through the large ward to the lavatory at the end of it? No special provision seems to be made for this apartment.

### BUILDINGS CONTEMPLATED AND IN PROGRESS.

Bromley.—Mr. Paul Waterhouse, M.A., F.R.I.B.A., was architect for the re-modelling and enlargement of Bromley Cottage Hospital. It was opened on the 6th inst. by the Dowager Countess of Derby in the presence of the Mayor (Alderman T. Davis, J.P.), and the Town Council.

Colchester.—Tenders are invited for the erection of several detached buildings at Mile End, Colchester, on behalf of the Essex County Lunatic Asylum Committee. Quantities may be had from the Clerk on deposit of £50 and a £5,000 security is required for the due performance of the contract.

Devizes.—A competition for an addition to the Devizes Cottage Hospital is being held; designs had to be sent in by the 10th inst., and the author of the design selected will be employed to carry out the work, while the author of the second design will receive a solatium of £10 10s. The committee has been unable to conform with the regulations of the Royal Institute of British Architects as to competitions.

Dewsbury.—Alterations and additions are contemplated to the hospital on behalf of the Joint Hospital Board Managers.

Market Drayton (Salop).—Mr. George A. Craig, of Market Drayton, is acting as architect for the erection of an operating-room, recovery ward, etc., at the Cottage Hospital. Tenders have just been delivered for the works.

Mansfield.—The Board of Management contemplate an enlargement of the hospital; £10,000 is the probable cost.

Terms have been agreed on for the amalgamation of the Royal Institute of British Architects and the Society of Architects to unite the profession in an attempt to carry through Parliament a Bill for the registration of architects.

### COMING EVENTS OF THE WEEK.

[Communications for this column must be sent to 29 Southampton Street, Strand, before noon on Wednesday.]

SATURDAY, MAY 13.—West London Hospital, Hammersmith Road. Demonstration of cases in wards, by Medical Registrar, 10 A.M.

MONDAY, MAY 15.—West London Hospital, Hammersmith Road. Demonstration by Surgical Registrar, 10 A.M.; Pathological Demonstration, 12 noon; Clinical Pathology, by Dr. Bernstein, 5 P.M.

TUESDAY, MAY 16.—West London Hospital, Hammersmith Road. Demonstration of Minor Operations, 11.30 A.M.; Lecture, "Head Injuries," by Mr. Armour, 5 P.M.

National Hospital, Queen's Square, W.C. Lecture, "Muscular Atrophy," by Dr. James Taylor, 3.30 P.M.

Central London Throat Hospital, Gray's Inn Road. Lecture, "Nose," by Dr. James Atkinson, 3.45 P.M.

City of London School, Victoria Embankment, E.C. Lecture, "Some Previous Epidemics of Measles," by F. M. Sandwith, M.D., 6 P.M.

London Orphan Asylum, Watford. Ninety-eighth Annual Festival Dinner, Whitehall Rooms, Hôtel Métropole. D. J. Morgan, J.P., in the chair, 7 P.M.

WEDNESDAY, MAY 17.—West London Hospital, Hammersmith Road. Gynæcological Demonstration, 10 A.M.; Lecture, "Head Injuries," by Mr. Armour, 5 P.M.

Princess Louise (Duchess of Argyll) opens the Grand Dutch Bazaar in aid of the new Ealing Hospital, Ealing Town Hall, 3 P.M.

City of London School, Victoria Embankment, E.C. Lecture, "Present Knowledge of Measles," by F. M. Sandwith, M.D., 6 P.M.

THURSDAY, MAY 18.—West London Hospital, Hammersmith Road. Demonstration by Surgical Registrar, 10 A.M.; Lecture, "Practical Medicine," by Dr. G. Stewart, 12.15 P.M.; Lecture, "Delusional Insanity," by Dr. Cole, 5 P.M.

Grand Dutch Bazaar (second day), in aid of new Ealing Hospital. Opening by Lady George Hamilton, Ealing Town Hall, 2.30 P.M.

City of London School, Victoria Embankment, E.C. Lecture, "The Black Death," by F. M. Sandwith, M.D., 6 P.M.

FRIDAY, MAY 19.—National Hospital, Queen's Square, W.C. Lecture, "Muscular Atrophy," by Dr. James Taylor, 3.30 P.M.

Central London Throat Hospital, Gray's Inn Road. Lecture, "Nose," by Dr. James Atkinson, 3.45 P.M.

West London Hospital, Hammersmith Road. Clinical Lecture, with cases, by Dr. Saunders, 5 P.M.

London Homœopathic Hospital, Great Ormond Street, W.C. Lecture, "Psychasthenia and Neurasthenia," by G. S. Goldsbrough, M.D., 5 P.M.

City of London School, Victoria Embankment, E.C. Lecture, "The Plague of To-day," by F. M. Sandwith, M.D., 6 P.M.

### APPOINTMENTS VACANT.

FULL particulars of these vacancies can be obtained on reference to our advertisement columns :

BECKETT HOSPITAL, BARNSLEY.—Second House Surgeon.

LEICESTER INFIRMARY.—Second House Physician.

MANCHESTER ROYAL INFIRMARY (The Convalescent Hospital, Cheadle).—Assistant Medical Officer.

ROYAL ALBERT HOSPITAL, DEVONPORT.—Assistant Resident Medical Officers.

ST. BARTHOLOMEW'S HOSPITAL, ROCHESTER.—Non-Resident Secretary to the House Committee.

WOLVERHAMPTON AND STAFFORDSHIRE GENERAL HOSPITAL.—House Surgeon.

